# Study on Microstructural Evolution, Strengthening and Deformation Mechanisms of a CoCrNi-Based Medium-Entropy Alloy with Different Annealing Times

**DOI:** 10.3390/ma19050945

**Published:** 2026-02-28

**Authors:** Pu Zhang, Chunjiang Zhao, Ruirui Wang, Yongping Liu, Yongan Chen

**Affiliations:** 1 School of Intelligent Manufacturing Industry, Shanxi University of Electronic Science and Technology, Linfen 041000, China; zhangpu@sxdzkj.edu.cn (P.Z.); zhaochunjiang@sxdzkj.edu.cn (C.Z.); wangruirui@sxdzkj.edu.cn (R.W.); liuyongping@sxdzkj.edu.cn (Y.L.); 2 School of Mechanical Engineering, Taiyuan University of Science and Technology, Taiyuan 030024, China; 3 School of Semiconductor and Physics, North University of China, Taiyuan 030051, China

**Keywords:** medium-entropy alloy, annealing time, strengthening mechanism, deformation mechanism

## Abstract

This study systematically investigates the influence of annealing time on the microstructure and mechanical properties of a (CoCrNi)_93.5_Al_3_Ti_3_C_0.5_ medium-entropy alloy. Following hot-rolling, the alloy was subjected to annealing treatments at 900 °C for 10 min (HA900-10) and 60 min (HA900-60). Microstructural characterization revealed that both alloys contained three types of precipitates: intergranular M_23_C_6_ and MC-type carbides, as well as γ′ phase. The HA900-10 specimen exhibited a low degree of recrystallization, whereas prolonged annealing promoted partial recrystallization, leading to the formation of a slightly heterogeneous structure (HA900-60). Additionally, the extended annealing facilitated the intragranular precipitation of nanoscale γ′ phase. Room-temperature tensile tests demonstrated that the HA900-10 and HA900-60 specimens achieved yield strengths of 1276 MPa and 1202 MPa, with total elongations reaching 26% and 28%, respectively. Quantitative strengthening analysis indicated that the strength of HA900-10 primarily originated from dislocation and grain boundary strengthening. For HA900-60, an additional significant contribution arose from the dislocation shearing mechanism induced by the intragranular γ′ precipitates. Analysis of the deformation mechanisms revealed that planar slip, assisted by the formation of stacking faults, dominated the room-temperature deformation, thereby ensuring sustained work-hardening capacity. This research provides a theoretical foundation for tailoring the microstructure and properties of multi-phase medium-entropy alloys through annealing process control.

## 1. Introduction

The pursuit of advanced metallic materials with superior mechanical properties represents a fundamental and ongoing objective in materials science, fueled by the stringent requirements of modern engineering [1,2,3]. In this endeavor, high- and medium-entropy alloys (H/MEAs), or multi-principal element alloys, have introduced a transformative alloy design strategy that departs from conventional single-principal-element frameworks [4,5,6]. These alloys are distinguished by their high configurational entropy and the incorporation of multiple principal elements, which often stabilize simple solid solution phases like face-centered cubic (FCC) structures and can lead to an exceptional blend of fracture toughness, corrosion resistance, and thermal stability [7].

Within this class of materials, the equiatomic CoCrNi MEA has been extensively studied as a model system due to its remarkable combination of damage tolerance and ductility [8]. This performance stems from a hierarchical deformation response, where mechanisms such as dislocation glide and nano-twinning are sequentially activated with increasing strain [9]. A persistent limitation, however, is its relatively moderate yield strength, typically around 300–500 MPa in the annealed state, restricting its use in structural applications [10]. Enhancing the yield strength of the CoCrNi system without severe compromise to its ductility therefore remains a critical goal.

The introduction of nanoscale precipitates offers a potent pathway to strength enhancement. Alloying the CoCrNi matrix with elements like Al and Ti can promote the formation of L1_2_-ordered γ′ precipitates, which effectively strengthen the alloy by interacting with dislocations [11,12,13]. Concurrently, the addition of interstitial carbon (C) contributes to solid solution strengthening and can facilitate the precipitation of various carbides, providing an additional strengthening mechanism. The interplay between the precipitation and recrystallization behavior during thermomechanical processing leads to the final microstructure and properties [14]. This is highly sensitive to heat treatment parameters. Specifically, annealing time is a pivotal variable that directly governs dislocation recovery, recrystallization fraction, grain size, and the morphology, size, and distribution of precipitates, thereby dictating the ultimate strength–ductility balance [15,16].

Recent research has often focused on employing complex multi-step heat treatments to engineer microstructures for property enhancement [17]. In contrast, we systematically investigated the influence of the annealing time on the microstructural evolution and mechanical properties of the CoCrNi base alloy. Such a time-centric study minimizes confounding factors, providing more direct insight into the competition and synergy between recrystallization and precipitation kinetics. This approach can yield fundamental guidance for tailoring the microstructure of such multi-component alloys.

Motivated by this rationale, the present study investigates a (CoCrNi)_93.5_Al_3_Ti_3_C_0.5_ (at.%) alloy subjected to hot-rolling followed by annealing. The annealing temperature was selected as 900 °C based on previous research experience [18], as it lies within a temperature window where both recrystallization and precipitation can occur simultaneously. A short annealing time (10 min) was chosen to retain deformation structures, while a long annealing time (60 min) was selected to allow partial recrystallization and sufficient precipitation without excessive grain growth. We systematically compare the microstructural features—including recrystallization completeness, grain structure, and the precipitation behavior between the two conditions. The correlation between these microstructural differences and the resulting room-temperature tensile properties is established. Furthermore, the dominant strengthening mechanisms are quantitatively assessed, and the underlying deformation mechanisms are elucidated through detailed post mortem microscopy. This work aims to provide theoretical and practical insights for optimizing the performance of such multi-phase MEAs through precise control of annealing treatment.

## 2. Experimental Methodology

An alloy with the nominal composition (CoCrNi)_93.5_Al_3_Ti_3_C_0.5_ (at.%) was synthesized via vacuum induction melting using high-purity raw materials (each >99.9%). Following melting under an inert atmosphere, the melt was cast into a mold and allowed to solidify inside the furnace. The as-cast ingot was homogenized at 1200 °C for 4 h to eliminate compositional inhomogeneities. It was then hot-rolled at 1100 °C in 5 passes, achieving an overall thickness reduction of approximately 95%. The rolling operation was performed in ambient air, and the rolled sheets were immediately quenched in water. The rolled sheets were then subjected to isothermal annealing at 900 °C for durations of 10 min and 60 min, respectively. All thermomechanical processes were carried out in air and terminated by water quenching. The annealed conditions are hereafter designated as HA900-10 and HA900-60 for brevity.

Quasi-static uniaxial tensile tests were conducted at ambient temperature (298 K) using an AG-X PLUS universal testing machine (SHIMADZU Corporation, Kyoto, Japan). Dog-bone-shaped specimens with a gauge section of 25 mm in length and 6 × 3 mm^2^ in cross-sectional area were machined. The gauge surfaces were ground with SiC paper prior to testing. A minimum of three tests were performed for each condition to ensure reliability. Tensile loading was applied parallel to the rolling direction at a constant strain rate of 1 × 10^−3^ s^−1^.

Crystal structure analysis was carried out using X-ray diffraction (XRD) on a SmartLab-3 KW diffractometer (Rigaku Corporation, Tokyo, Japan) with Cu Kα radiation. Diffraction patterns were recorded over a 2θ interval of 20–90° with a step increment of 0.02°. A SIGMA500 field-emission scanning electron microscope (SEM) (Carl Zeiss AG, Oberkochen, Germany) equipped with an Oxford Nordlys Max3 electron backscatter diffraction (EBSD) detector (Oxford Instruments plc, Abingdon, UK) was used for microstructural observations. Fine-scale features were examined with a Tecnai G2 F30 S-TWIN transmission electron microscope (TEM) (FEI Company, Hillsboro, OR, USA) coupled with an energy-dispersive X-ray spectroscopy (EDS) system. EBSD mapping was performed with a step size of 0.05 µm on specimens cut parallel to the rolling direction. Specimens for SEM and EBSD were prepared by electro-phishing in a solution of 90% ethanol (Sigma-Aldrich, Munich, Germany) and 10% perchloric acid (Sigma-Aldrich, St. Louis, MO, USA) (by volume) at 15 V and 273 K. TEM foils were prepared by mechanically thinning to approximately 50 µm, punching 3 mm disks, and final thinning via ion milling at room temperature.

## 3. Results and Discussion

### 3.1. Microstructural Evolution

**Figure 1:**
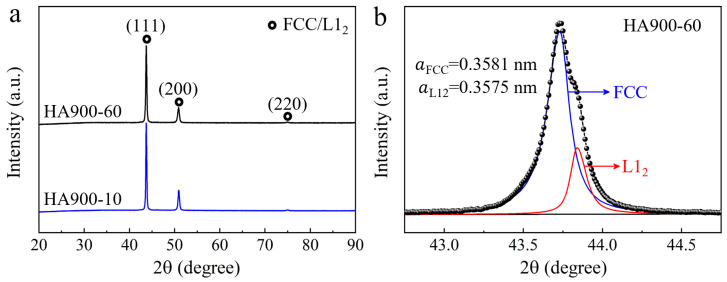
(**a**) XRD patterns of the HA900-10 and HA900-60 specimens; (**b**) peak deconvolution of the (200) diffraction peak for the HA900-60 specimen, identifying the γ and γ′ phases.

X-ray diffraction patterns obtained from the HA900-10 and HA900-60 specimens are shown in Figure 1a. Both patterns contain reflections characteristic of a face-centered cubic (FCC) structure, indicating that the γ matrix phase is preserved after annealing. A noticeable asymmetry is observed in all major diffraction peaks of the HA900-60 specimen, suggesting the existence of a secondary phase with a lattice similar to that of the matrix. To verify this, the (200) peak of HA900-60 was deconvoluted (Figure 1b), revealing two distinct contributions corresponding to the γ matrix and the γ′ precipitate phase. The lattice parameters were determined as 0.3581 nm for the γ phase and 0.3575 nm for the γ′ phase. In contrast, no peak asymmetry is observed for the HA900-10 specimen (Figure S1), suggesting that either the γ′ phase did not precipitate or its volume fraction was below the detection limit of XRD due to the short annealing time (10 min), which may have been insufficient for appreciable precipitation. Furthermore, no characteristic peaks corresponding to other precipitated phases are detected, likely due to their low volume fractions, necessitating more sensitive characterization techniques for confirmation.

**Figure 2:**
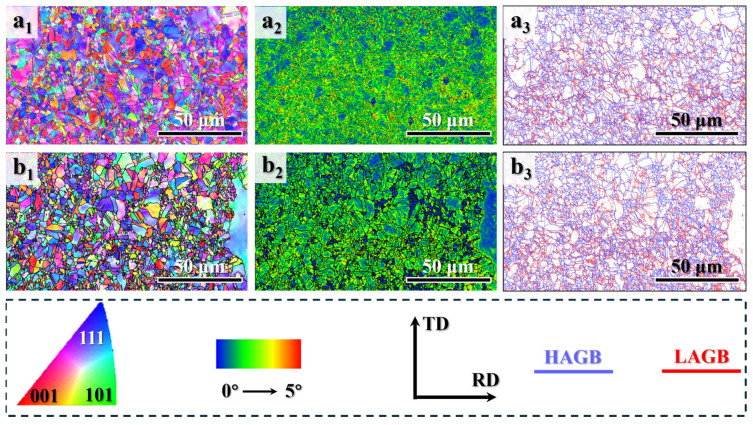
EBSD analysis of the HA900-10 (**a_1_**–**a_3_**) and HA900-60 (**b_1_**–**b_3_**) specimens: (**a_1_**,**b_1_**) inverse pole figure maps; (**a_2_**,**b_2_**) kernel average misorientation maps; (**a_3_**,**b_3_**) grain boundary maps (blue and red lines represent low-angle and high-angle grain boundaries, respectively).

**Figure 3:**
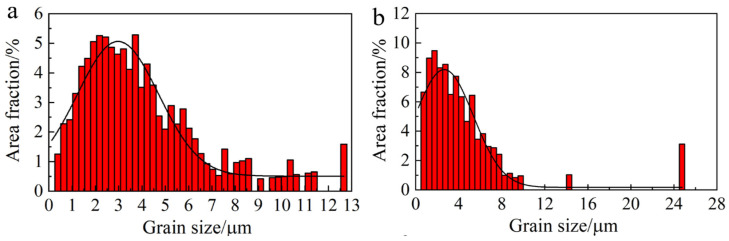
Histogram of grain size distribution fitted with a Gaussian function for the (**a**) HA900-10 and (**b**) HA900-60 specimens.

The grain structures were further investigated using EBSD. As shown in the inverse pole figure map (IPF, Figure 2(a_1_)), the HA900-10 specimen consists of relatively fine grains with a uniform size distribution. A strong <111> and <001> texture is evident, inherited from the prior hot-rolling deformation. The corresponding kernel average misorientation (KAM) map (Figure 2(a_2_)) and grain boundary map (Figure 2(a_3_)) reveal a high density of geometrically necessary dislocations (GNDs) and a significant fraction of low-angle grain boundaries (LAGBs), respectively, within the grains. This typically indicates a low degree of recrystallization. Upon extending the annealing time to 60 min (HA900-60 specimen), the preferential texture is essentially eliminated, giving way to a random grain orientation (Figure 2(b_1_)). The microstructure evolves into a blend of partially coarsened grains and refined grains, demonstrating a certain level of heterogeneity. The KAM map (Figure 2(b_2_)) and grain boundary map (Figure 2(b_3_)) show a slightly lower density of GNDs and LAGBs in the HA900-60 specimen compared to HA900-10. Statistical analysis of grain size distributions (Figure 3a,b) yields average grain sizes of 4.03 ± 1.1 μm and 4.39 ± 1.2 μm for the HA900-10 and HA900-60 specimens, respectively. By comparing the tailing phenomenon of the Gaussian fitted curves, it can be inferred that the grain size heterogeneity of HA900-60 is greater than that of the HA900-10 specimen.

**Figure 4:**
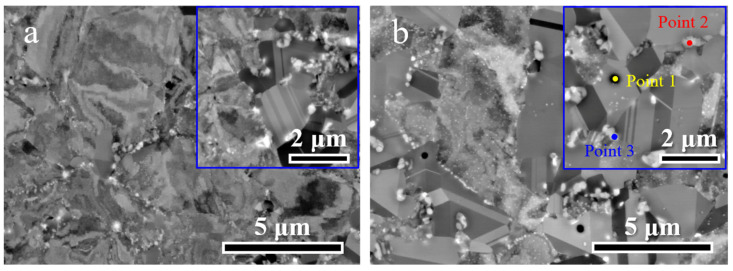
BSE micrographs of the (**a**) HA900-10 and (**b**) HA900-60 specimens. Insets are higher-magnification views showing precipitates distributed along grain boundaries.

Microstructural morphology was examined using backscattered electron (BSE) imaging. Figure 4a shows that the grains in the HA900-10 specimen contain abundant deformation structures that are not fully recovered, consistent with the low recrystallization degree indicated by EBSD. In contrast, some grains in the HA900-60 specimen (Figure 4b) exhibit a “clean” appearance free of deformation features, signifying completion of recrystallization. A key observation is the presence of finely dispersed precipitates within the incompletely recrystallized regions of the HA900-60 specimen, while this is absent in HA900-10. Enlarged insets in both figures clearly show three types of particles with black, gray, and white contrasts distributed along grain boundaries. Comparison reveals a slight coarsening of these precipitates in the HA900-60 specimen. EDS point analysis at the marked locations in Figure 4b indicates that the black particles are enriched in Ti, while the gray and white particles are rich in Cr and Ni (Figure S2), respectively. Given the concurrence of recrystallization and precipitation in this study, it can be deduced that the absence of significant grain coarsening in the HA900-60 specimen is primarily attributed to the Zener pinning effect exerted by the intergranular precipitates, which effectively hinders grain boundary migration and grain growth.

**Figure 5:**
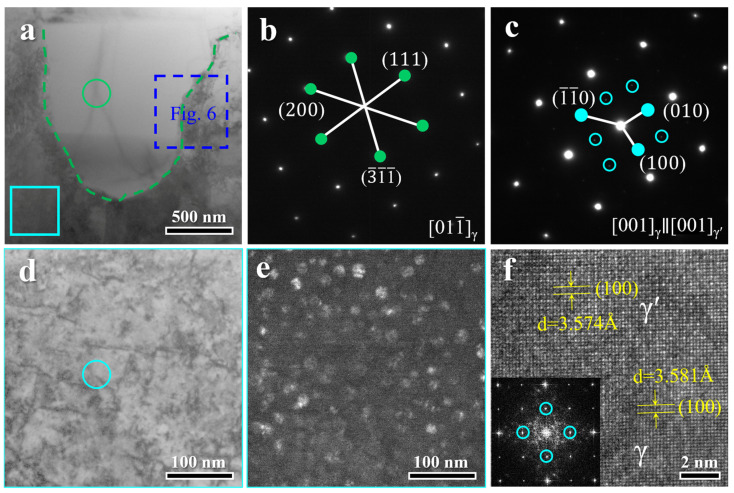
TEM analysis of the HA900-60 specimen: (**a**) BF image showing a fully recrystallized region (green dashes) and an incompletely recrystallized region (cyan rectangle); (**b**,**c**) SAED patterns corresponding to the regions in (**a**); (**d**) high-magnification view of the incompletely recrystallized area showing dislocations; (**e**) DF image taken using the g = (001) superlattice spot, highlighting the γ′ phase; (**f**) HRTEM and FFT images showing the γ/γ′ interface.

TEM was employed to resolve the fine-scale microstructure of the HA900-60 specimen. The bright-field (BF) image in Figure 5a shows a fully recrystallized grain (outlined by green dashes), whose corresponding selected-area electron diffraction (SAED) pattern (Figure 5b) is indexed to the γ matrix. The region enclosed by the cyan rectangle represents an incompletely recrystallized area, where residual dislocations are visible at higher magnification (Figure 5d). SAED analysis of this region (Figure 5c) reveals superlattice spots, confirming the presence of the γ′ phase. A dark-field (DF) image taken using the g = (001) superlattice spot (Figure 5e) vividly displays these γ′ nanoparticles. The high-resolution TEM (HRTEM) image and its Fast Fourier transform (FFT) pattern in Figure 5f further verify the γ′ phase. The lattice parameters calculated by directly measuring interplanar spacings are 0.3581 nm and 0.3574 nm for the γ and γ′ phases, respectively, in excellent agreement with the XRD results. Based on the above characterization results, it can be inferred that the particles observed in the incompletely recrystallized regions of the HA900-60 specimen shown in Figure 4b are intragranular γ′ precipitates. These precipitates hinder the movement and recovery of dislocations, which is the primary reason for the incomplete recrystallization of these grains. The absence of an intragranular γ′ phase in the HA900-10 specimen is likely attributed to insufficient annealing time for the nucleation and growth of γ′ precipitates, which is consistent with the precipitation kinetics reported in similar CoCrNi-based alloys [13,18,19,20].

**Figure 6:**
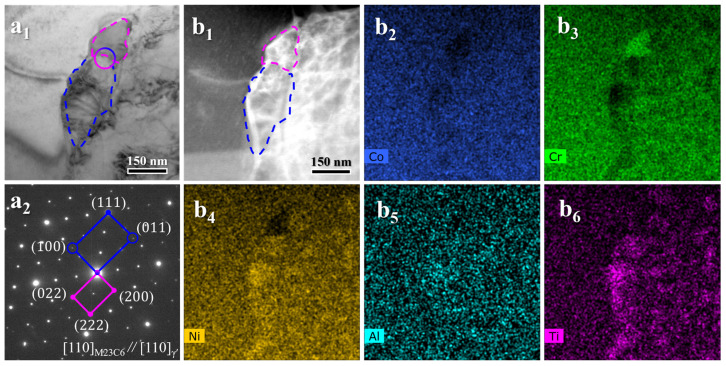
Detailed analysis of intergranular precipitates in the HA900-60 specimen: (**a_1_**) high-magnification BF image of a grain boundary region; (**a_2_**) indexed SAED pattern; (**b_1_**–**b_6_**) EDS maps showing the distribution of constituent elements.

**Figure 7:**
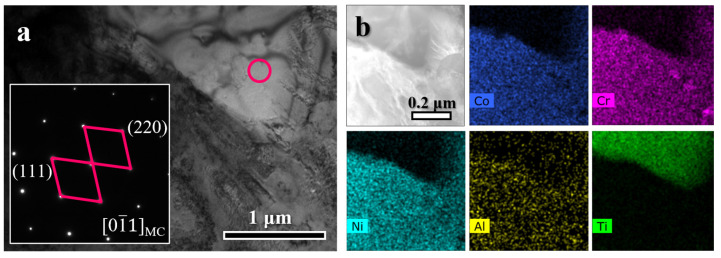
Characterization of an MC-type carbide in the HA900-60 specimen: (**a**) BF image and SAED pattern; (**b**) EDS maps showing the distribution of constituent elements.

A detailed examination of the area (Figure 6(a_1_)) within the blue dashed box in Figure 5a, combined with EDS mapping (Figure 6(b_1_–b_6_)), identifies two types of precipitates at a grain boundary. The precipitate marked by the pink dashed line is enriched in Cr, while that within the blue dashed line is rich in Ni, Ti, and Al. Correlated SAED indexing (Figure 6(a_2_)) confirms them as M_23_C_6_-type carbide and γ′ phase, respectively. They maintain a clear crystallographic orientation relationship: (111)M23C6//(111)γ′ and [110]M23C6//[110]γ′. A coarse particle is visible in the bright-field TEM image of Figure 7a. Energy-dispersive X-ray spectroscopy mapping (Figure 7b) demonstrates that this particle is Ti-rich and depleted in Co, Cr, Ni, and Al. Selected-area electron diffraction (SAED) patterns indexed this phase as an MC-type carbide.

In summary, extending the annealing time from 10 min to 60 min at 900 °C promotes partial recrystallization, reduces dislocation density, and induces the intragranular precipitation of nanoscale γ′ phase. These time-dependent microstructural changes are key to understanding the subsequent mechanical behavior.

### 3.2. Room-Temperature Mechanical Properties

**Figure 8:**
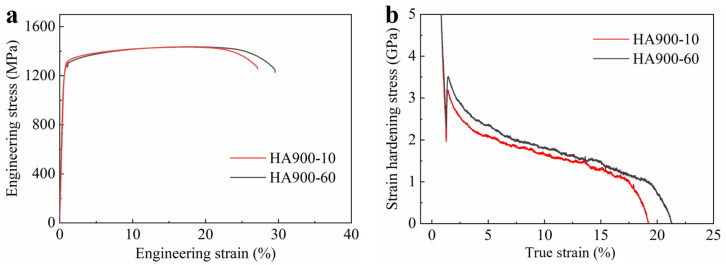
(**a**) Room-temperature engineering stress–strain curves of the HA900-10 and HA900-60 specimens; (**b**) corresponding true stress–strain curves and work-hardening rate curves.

Figure 8a displays the engineering stress–strain curves of both specimens at room temperature. Compared to the as-cast alloy (Figure S3), both specimens in this study exhibited significantly enhanced comprehensive mechanical properties. The HA900-10 specimen exhibits a yield strength of 1276 MPa, an ultimate tensile strength of 1434 MPa, and a total elongation of 26%. When the annealing time is extended to 60 min (HA900-60), the yield strength slightly decreases to 1202 MPa, while both the ultimate tensile strength (1436 MPa) and total elongation (28%) remain at excellent levels. It can be seen that with prolonged annealing, the strength of the alloy does not decrease significantly, while the elongation exhibits a slight improvement. The work-hardening rate curves shown in Figure 8b elucidate the underlying reason for this retained performance: the HA900-60 specimen demonstrates a higher work-hardening capacity during the mid-to-late stages of plastic deformation, effectively compensating for the potential strength loss caused by reduced dislocation density and partial grain coarsening. Additionally, the finely dispersed intragranular nano-sized γ′ precipitates in the HA900-60 specimen play a critical role in compensating for the loss of dislocation strengthening and maintaining high tensile ductility.

### 3.3. Quantitative Analysis of Strengthening Mechanisms

Although the HA900-10 and HA900-60 specimens exhibit comparable room-temperature yield strengths, their distinct microstructures suggest potentially different dominant strengthening mechanisms. Therefore, a quantitative assessment of the individual contributions to the yield strength was performed for both of them.

For the HA900-60 specimen, the yield strength ($\sigma_{Y}$) can be expressed as the sum of the intrinsic matrix strength ($\sigma_{I}$), solid solution strengthening ($\Delta \sigma_{S}$), dislocation strengthening ($\Delta \sigma_{D}$), grain boundary strengthening ($\Delta \sigma_{G}$), and Orowan strengthening from intergranular MC and M_23_C_6_ carbides, as well as γ′ phase ($\Delta \sigma_{p}^{o}$) and strengthening due to the shearing of intragranular γ′ precipitates ($\Delta \sigma_{p}^{s}$), as shown in Equation (1). The last term is omitted for the HA900-10 specimen, which lacks intragranular γ′ precipitates.

(1) $$\begin{array}{c} \sigma_{Y}=\sigma_{I}+\Delta \sigma_{S}+\Delta \sigma_{D}+\Delta \sigma_{G}+\Delta \sigma_{p}^{o}+\Delta \sigma_{p}^{s} \end{array}$$

The intrinsic strength of the CoCrNi matrix, $\sigma_{I}$, is taken as 216 MPa [20]. Considering that solid solution strengthening is not the primary mechanism in this alloy system, $\Delta \sigma_{S}=$ 86 MPa is adopted from reports on alloys with similar compositions for simplification [20,21].

**Figure 9:**
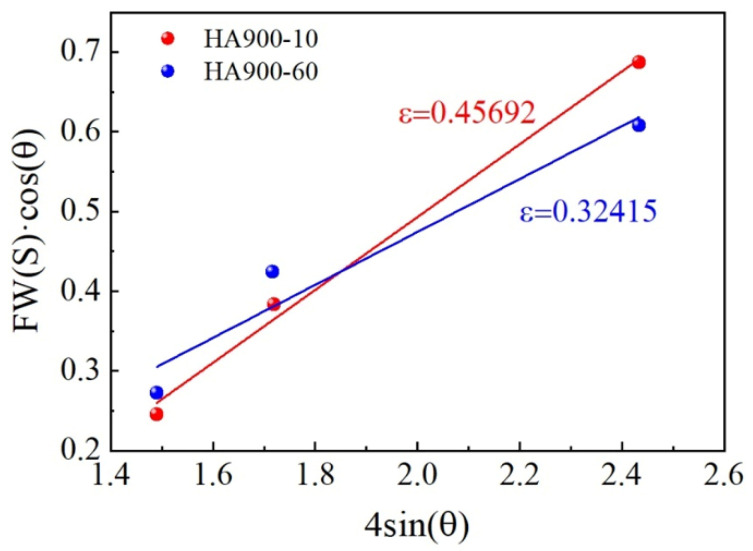
Linear fitting of XRD data based on the Williamson–Hall method for calculating micro-strain and dislocation density.

The KAM maps indicate a considerable population of residual dislocations in both specimens. These dislocations interact with newly generated dislocations during the initial stage of deformation, leading to tangles that impede slip. The dislocation strengthening contribution ($\Delta \sigma_{D}$) is calculated using the Bailey–Hirsch relationship (Equation (2)) [22].

(2) $$\begin{array}{c} \Delta \sigma_{D}=M\alpha Gb\rho^{\frac{1}{2}} \end{array}$$

where M is the Taylor factor (3.06), α is a constant (0.2), G is the shear modulus (87 GPa [23]), and b is the Burgers vector ($b=\sqrt{2}a/2$, with a= 0.3581 nm). The dislocation density ρ is determined from XRD data using the Williamson–Hall method (Equations (3) and (4)).

(3) $$\begin{array}{c} \rho =2\sqrt{3}\cdot \frac{\varepsilon }{Db} \end{array}$$

(4) $$\begin{array}{c} FW\left(S\right)\cdot \cos\theta =\frac{K\lambda }{D}+4\varepsilon \cdot \sin\theta \end{array}$$

where ε denotes the micro-strain of the material, D is the average grain size, $FW\left(S\right)$ indicates the peak broadening. K is a coefficient (taken as 0.9), λ is the wavelength of Cu Kα radiation (0.15405 nm), and θ is the Bragg angle corresponding to the XRD peak. By substituting the XRD data into Equation (4), a linear fitting of $FW\left(S\right)\cdot \cos\theta$ versus 4sinθ was performed, with the results presented in Figure 9. The micro-strain (ε) values obtained for specimens HA900-10 and HA900-60 were 0.45692 and 0.32415, respectively. Substituting these into Equation (3) yields dislocation densities of 1.5 × 10^15^ m^−2^ and 1.0 × 10^15^ m^−2^. These values were then used in Equation (2), giving dislocation strengthening contributions of 532 MPa and 435 MPa, respectively.

Grain boundaries act as barriers to dislocation motion, and their strengthening contribution $\Delta \sigma_{G}$ follows the Hall–Petch relationship (Equation (5)).

(5) $$\begin{array}{c} \Delta \sigma_{G}=kd^{-\frac{1}{2}} \end{array}$$

where k is the Hall–Petch coefficient (taken as 568 MPa·μm^1/2^ [24]) and d is the average grain size. Using the grain sizes from EBSD statistics, $\Delta \sigma_{G}$ is calculated to be 283 MPa and 271 MPa for HA900-10 and HA900-60, respectively.

Based on the precipitate size and volume fraction data summarized in Table 1 and Table 2, and neglecting the contribution from micron-sized MC carbides, the Orowan strengthening contribution $\Delta \sigma_{p}^{o}$ from nano-sized M_23_C_6_/γ′ clusters and submicron MC carbides is calculated using Equation (6). The values are 39 MPa and 61 MPa for the HA900-10 and HA900-60, respectively.

(6) $$\begin{array}{c} \Delta \sigma_{p}^{o}=0.538\frac{Gbf^{1/2}}{r}\ln\left(\frac{r}{2b}\right) \end{array}$$

where f and r denote the volume fraction and average particle diameter of the precipitates, respectively.

For the intragranular nano-sized γ′ precipitates in the HA900-60 specimen, the dominant interaction with dislocations is shearing. The resulting strengthening $\Delta \sigma_{p}^{s}$ is determined by the maximum value among coherent strengthening ${\Delta \sigma }_{CS}$, modulus mismatch strengthening $\Delta \sigma_{MS}$, and order strengthening ${\Delta \sigma }_{OS}$.

(7) $$\begin{array}{c} {\Delta \sigma }_{CS}=M\cdot \alpha_{\varepsilon }{\left(G\cdot \delta \right)\;}^{3/2}{\left(\frac{rf}{0.5Gb}\right)}^{1/2} \end{array}$$

(8) $$\begin{array}{c} \Delta \sigma_{MS}=M\cdot 0.0055{\left(\Delta G\right)}^{3/2}{\left(\frac{2f}{G}\right)}^{1/2}{\left(\frac{r}{b}\right)}^{3m/2-1} \end{array}$$

(9) $$\begin{array}{c} {\Delta \sigma }_{OS}=M\cdot 0.81\frac{\gamma_{APB}}{2b}{\left(\frac{3\pi f}{8}\right)}^{1/2} \end{array}$$

Calculations using Equations (7)–(9) [25] with parameters from Table 2 and known constants (lattice misfit δ, shear modulus difference ΔG= 10 GPa [23], anti-phase boundary energy $\gamma_{APB}$ = 200 mJ/m^2^ [26], constant m = 0.85. The $\Delta \sigma_{p}^{s}$ of the HA900-60 specimen was calculated to be 180 MPa.

**Figure 10:**
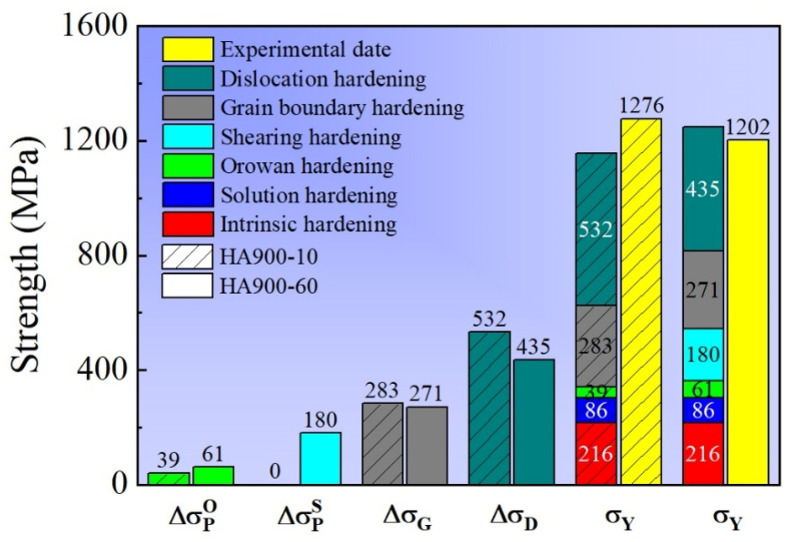
Decomposition of room-temperature yield strength contributions for the HA900-10 and HA900-60 specimens.

The distribution of individual strengthening contributions to the yield strength is summarized in Figure 10. The calculated values show good agreement with experimental measurements. The analysis reveals that the strength of the HA900-10 specimen originates primarily from dislocation and grain boundary strengthening. For the HA900-60 specimen, in addition to these two mechanisms, the shearing of coherent intragranular γ′ precipitates emerge as a crucial third strengthening source. It is noted that the trend in the calculated yield strength values for the two samples contradicts the experimental results. This discrepancy may arise because the intragranular γ′ phase in the HA900-60 specimen is primarily distributed within the incompletely recrystallized grains, rather than uniformly throughout the entire alloy microstructure. Consequently, the shearing hardening value calculated using the existing formula is overestimated.

### 3.4. Deformation Mechanisms

**Figure 11:**
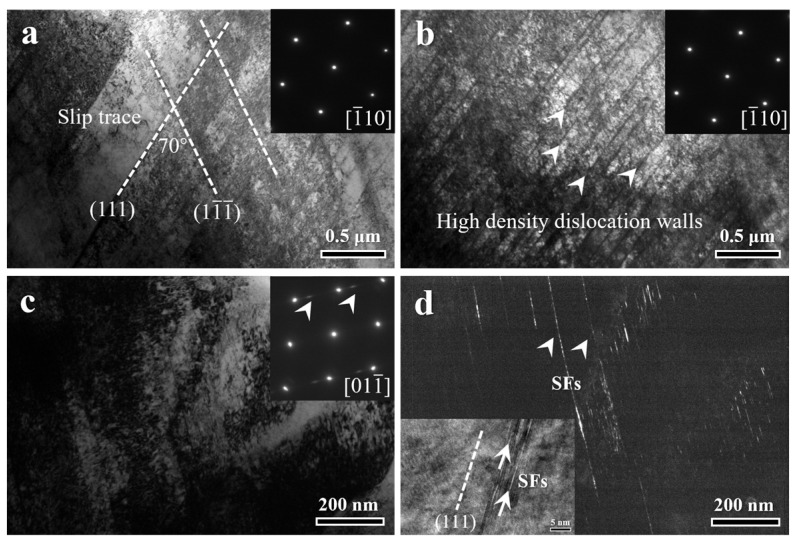
TEM micrographs of the HA900-10 specimen after room-temperature tensile deformation: (**a**) planar slip features in a low-strain region; (**b**) high-density dislocation walls in a high-strain region; (**c**) bright-field image showing deformation-induced stacking faults and the corresponding diffraction pattern; (**d**) dark-field image of a stacking fault region and an HRTEM image showing stacking fault bundles.

To elucidate the deformation mechanisms, TEM observations were conducted on the HA900-10 specimen after tensile fracture. TEM examination of the deformed HA900-10 specimen revealed planar slip bands on {111} planes, intersecting at approximately 70° in regions subjected to low strain (Figure 11a). This slip morphology is typical of FCC alloys with low stacking fault energy (SFE), where dislocations tend to glide in planar configurations or dissociate into Shockley partials, hindering cross-slip. The presence of interstitial carbon atoms further elevates lattice friction, thereby reinforcing the tendency for planar slip [27]. In the high-strain region near the fracture (Figure 11b), the {111} slip bands refine, forming high-density dislocation walls with spacings of about 10–100 nm (white arrows). This significantly reduces the mean free path of dislocations, contributing to enhanced work hardening. Figure 11c shows the deformed microstructure in the fracture region, and its corresponding diffraction pattern exhibits streaks, confirming the presence of deformation-induced stacking faults. The dark-field image in Figure 11d reveals the morphology of SFs. The HRTEM inset shows that SFs pile up on adjacent {111} planes, forming stacking fault bundles about 5 nm thick. SFs on {111} planes are typically generated by the dissociation of perfect dislocations with a Burgers vector of 1/2 <110> into 30° and 90° Shockley partials [28]. SFs play a dual role in plastic deformation: on one hand, the glide of partials at SF termini accommodates plastic strain; on the other hand, the unique atomic arrangement within SFs increases the resistance to dislocation passage, thereby contributing to work hardening. In summary, the room-temperature deformation of the HA900-10 specimen is dominated by planar dislocation slip, assisted by stacking faults. In the early stages of deformation, work hardening arises mainly from dislocation–dislocation interactions. During the mid-to-late stages, the dynamically refined slip bands and the complex interactions among dislocations, dislocation walls, and stacking faults become the primary work-hardening mechanisms.

## 4. Conclusions

The present work systematically evaluates how variations in annealing duration affect the microstructural evolution and resulting mechanical behavior of a (CoCrNi)_93.5_Al_3_Ti_3_C_0.5_ medium-entropy alloy. The findings demonstrate that annealing time critically governs the interplay between recrystallization and precipitation processes, offering a pathway to engineer microstructures with optimized strength–ductility combinations.

(1) After short-time annealing (10 min), the microstructure exhibits incomplete recrystallization with retained high dislocation density and fine grains. In contrast, prolonged annealing (60 min) promotes partial recrystallization and stimulates the precipitation of nano-sized γ′ phase within the grains. Both of the specimens show the presence of M_23_C_6_, MC-type carbides and γ′ phase distributed along grain boundaries.

(2) The strength of the HA900-10 alloy primarily originates from dislocation strengthening and grain refinement. For HA900-60, in addition to these mechanisms, strengthening via shearing of the γ′ precipitates are introduced, compensating for the potential strength loss due to reduced dislocation density, thereby maintaining a high yield strength (1202 MPa) while retaining excellent elongation (28%).

(3) During deformation, planar slip combined with stacking faults jointly governs the work-hardening behavior of the alloy. This research demonstrates that by controlling annealing time, the coordination between recrystallization and precipitation processes in multi-phase medium-entropy alloys can be achieved, offering important guidance for designing novel structural materials with both high strength and high ductility.

## Figures and Tables

**Table 1:** Volume fractions and average particle sizes of precipitates in the HA900-10 sample.

Precipitates	Volume Fraction (%)	Average Particle Size (nm)
M_23_C_6_ + Intergranular γ′	0.21 ± 0.02	101 ± 2.68
MC	0.17 ± 0.01	313 ± 4.11

**Table 2:** Volume fractions and average particle sizes of precipitates in the HA900-60 sample.

Precipitates	Volume Fraction (%)	Average Particle Size (nm)
M_23_C_6_ + Intergranular γ′	1.14 ± 0.09	148 ± 2.94
MC	0.24 ± 0.02	316 ± 4.25
Intragranular γ′	3.8 ± 0.7	18 ± 0.5

## Data Availability

The original contributions presented in this study are included in the article/Supplementary Materials. Further inquiries can be directed to the corresponding author.

## Supplementary Materials

The following supporting information can be downloaded at: https://www.mdpi.com/article/10.3390/ma19050945/s1, Figure S1. Magnified view of the XRD pattern showing the (200) peak of the HA900-10 specimen; Figure S2. EDS point analysis: (a), (b) and (c) correspond to the points 1, 2, and 3 at the marked locations in Figure 4b, respectively; Figure S3. Room-temperature engineering stress-strain curves of the as-cast alloy.
