# Supplementary material for: Study on Microstructural Evolution, Strengthening and Deformation Mechanisms of a CoCrNi-Based Medium-Entropy Alloy with Different Annealing Times
Source: Materials (Basel). 2026 Feb 28;19(5):945. doi: 10.3390/ma19050945 (PMC12986024; doi:10.3390/ma19050945)
Supplement: Supplementary file 1 [file materials-19-00945-s001.zip › materials-4160659-supplementary.pdf]

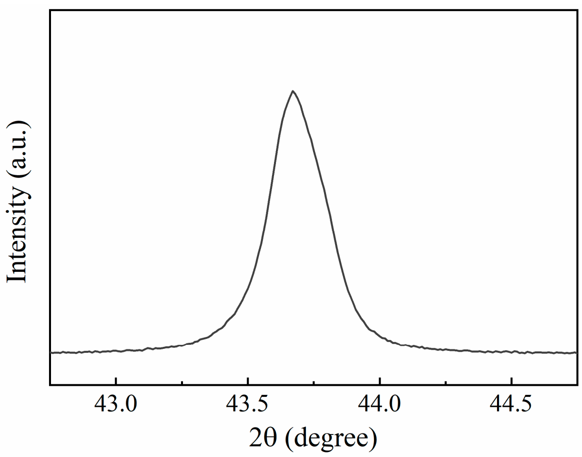

Figure S1. Magnified view of the XRD pattern showing the (200) peak of the HA900-10 specimen.

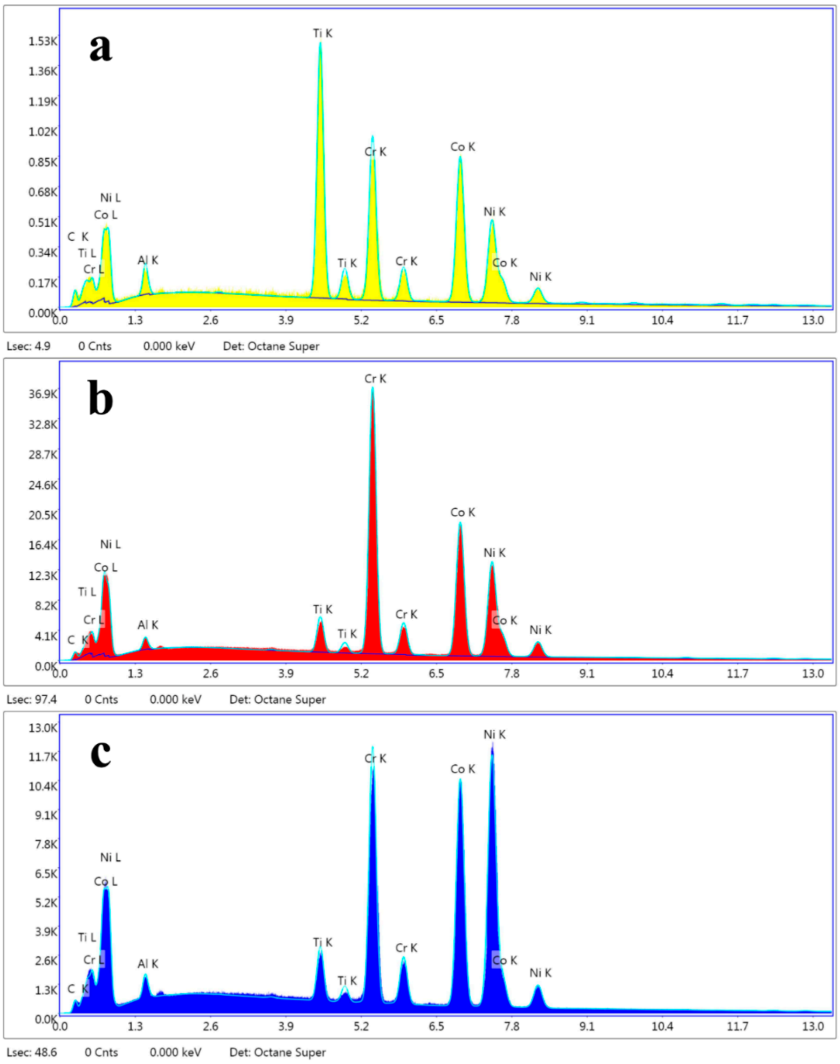

Figure S2. EDS point analysis: (a), (b) and (c) correspond to the points 1, 2, and 3 at the marked locations in Figure 4b, respectively.

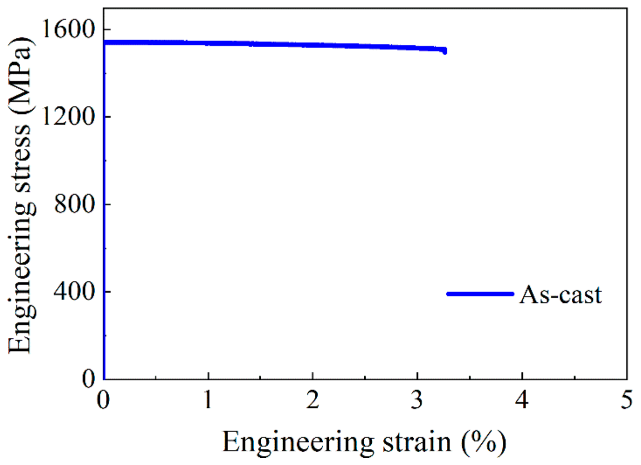

Figure S3. Room-temperature engineering stress-strain curves of the as-cast alloy.
